# Teachers’ Self-Efficacy in Dyscalculia: Development and Psychometric Validation of a New Scale

**DOI:** 10.3390/jintelligence14030050

**Published:** 2026-03-18

**Authors:** Gülçin Oflaz, Kübra Polat, Yılmaz Mutlu, Zekeriya Çam

**Affiliations:** 1 Department of Mathematics and Science Education, Faculty of Education, Sivas Cumhuriyet University, 58140 Sivas, Türkiye; kubraapolaat@hotmail.com.tr; 2 Faculty of Education, Muş Alparslan University, 49250 Muş, Türkiye; y.mutlu@alparslan.edu.tr (Y.M.); z.cam@alparslan.edu.tr (Z.Ç.)

**Keywords:** dyscalculia, teacher self-efficacy, scale development, mathematics learning difficulties, teacher competencies

## Abstract

The aim of this study is to develop a valid and reliable scale for measuring the self-efficacy of primary school and mathematics teachers regarding dyscalculia. Grounded in Bandura’s Social Cognitive Theory, the study followed established scale development procedures. In the initial phase, a pool of 42 items was generated to assess teachers’ self-efficacy regarding dyscalculia. The items were reviewed by a panel of seven experts in the fields of psychometrics, mathematics education, special education, and psychology to ensure content validity. Based on expert evaluations, four items were removed due to overly technical phrasing that could lead to misinterpretation, reducing the pool to 38 items. Subsequently, Exploratory Factor Analysis (EFA) conducted with 273 teachers indicated that four additional items exhibited inadequate factor loadings or problematic cross-loadings; these items were also excluded. The resulting Dyscalculia Self-Efficacy Scale (DSES) comprises 34 items organized into four factors: “Dyscalculia Symptoms”, “Providing Psychological Support to Children with Dyscalculia”, “Diagnosing Dyscalculia”, “Providing Support in the Teaching Process”. Confirmatory Factor Analysis conducted with a separate sample of 242 teachers yielded strong model fit indices, supporting the construct validity of the scale. The overall scale demonstrated high internal consistency (Cronbach’s α = 0.980, McDonald’s ω = 0.980). Correlation analyses with established instruments provided evidence of convergent and discriminant validity. The findings indicate that the DSES is a valid and reliable instrument for assessing teachers’ self-efficacy regarding dyscalculia.

## 1. Introduction

Teachers are among the most critical agents in the educational environment, as they guide and shape the learning process through their actions. These actions are influenced by their motivations, perceptions, beliefs, and experiences (Pajares, 1992). Consequently, teachers’ beliefs, perceptions, and motivations have a substantial impact on their instructional practices, thereby shaping the learning environment. In this regard, teachers’ self-efficacy beliefs are a determining factor in both their own professional effectiveness and their students’ academic success.

The role of primary school teachers is of paramount importance in identifying children with dyscalculia and in planning and implementing effective and appropriate interventions. During the transition from primary to middle school, the knowledge and awareness of middle school mathematics teachers—who continue to teach fundamental mathematical skills—regarding dyscalculia also play a vital role in this process. The first person to recognize the symptoms of dyscalculia is often the classroom teacher; therefore, assessing teachers’ self-efficacy regarding dyscalculia constitutes an important necessity. Enhancing teachers’ knowledge and intervention competencies is essential for supporting students with dyscalculia and improving the quality of their learning experiences.

Various methods and strategies exist for effectively teaching mathematics to children with dyscalculia. In this context, designing appropriate interventions tailored to students’ individual needs is of considerable importance. Research has demonstrated that students with dyscalculia can attain levels of achievement comparable to those of their peers when provided with appropriate support and intervention (Nelson & Powell, 2018). However, for this process to function effectively, teachers must possess sufficient awareness of dyscalculia and be competent in implementing evidence-based teaching methods and strategies for these students. Yet research indicates that teachers struggle to access appropriate interventions for children with dyscalculia (Chideridou–Mandari et al., 2016) and that their awareness and knowledge of dyscalculia remain insufficient (Chinn & Ashcroft, 2006; Dias et al., 2013; Mutlu et al., 2022). This situation impedes effective intervention planning and adversely affects instructional quality. Accordingly, raising teachers’ awareness of dyscalculia and equipping them with the competencies to plan, organize, and implement appropriate interventions can be regarded as a fundamental prerequisite for quality mathematics education.

Research demonstrates that teachers’ self-efficacy beliefs significantly influence their classroom attitudes and behaviors (Bandura, 1997; Cerit, 2011). Teachers with high self-efficacy tend to be less critical of students who make errors, are more likely to address students’ emotional and behavioral challenges and are less inclined to refer them for special education services (Poulou, 2007). Furthermore, self-efficacy is considered critical not only for goal attainment but also for guiding the process toward that goal (Bandura, 2012). In this context, assessing teachers’ self-efficacy regarding dyscalculia can be viewed as an important factor in supporting children with dyscalculia throughout their educational journey. Teachers’ self-efficacy in this domain is crucial for setting appropriate instructional goals and demonstrating effective performance in teaching students with dyscalculia. Moreover, high self-efficacy beliefs may help prevent unnecessary referrals to special education.

### 1.1. Teacher Self-Efficacy

According to Bandura’s Social Cognitive Theory, teacher self-efficacy refers to teachers’ beliefs in their capacity to influence students’ learning outcomes (Bandura, 1977; Guskey & Passaro, 1994). This theoretical framework positions self-efficacy as a salient indicator of effective teaching practices (Vatou et al., 2024). These beliefs guide teachers’ motivation, risk-taking, and persistence when confronting instructional challenges (Gabriele & Joram, 2007) and serve as key determinants of instructional quality and student learning (Guskey & Passaro, 1994; Skaalvik & Skaalvik, 2007).

Teachers with strong self-efficacy believe they can positively shape students’ learning through effective instruction. They are characterized by persistence, openness to innovation, and the use of diverse feedback strategies in their practices (Allinder, 1994; Gibson & Dembo, 1984; Gabriele & Joram, 2007; Guskey, 1998). Defined as “teachers’ beliefs about their ability to influence students’ learning outcomes, especially struggling or unmotivated students” (Guskey & Passaro, 1994, p. 628), self-efficacy is particularly vital when teaching students with learning difficulties or special educational needs (Brownell et al., 2006; Specht et al., 2016).

Teachers with high self-efficacy have been shown to enhance student achievement and motivation (Alexander, 2020; Engin, 2020; Caprara et al., 2006; Gibson & Dembo, 1984; Guo et al., 2011; Mojavezi & Tamiz, 2012; Hendricks et al., 2024; Morris et al., 2017). They demonstrate effectiveness in classroom management, willingness to adapt instruction for diverse learners, and a capacity to foster positive classroom climates (Aloe et al., 2014; Brouwers & Tomic, 2000; Friedman & Kass, 2002; Holzberger et al., 2013; Hsiao et al., 2011; Krasniqi & Ismajli, 2022; Miller et al., 2017; Tschannen-Moran & Woolfolk Hoy, 2001). Consequently, teacher self-efficacy influences not only students’ achievement and motivation but also their attitudes and their own self-efficacy beliefs (Tschannen-Moran & Woolfolk Hoy, 2001).

Furthermore, teachers with high self-efficacy assume responsibility for student outcomes, provide constructive feedback, and design effective learning experiences. They encourage student autonomy while reducing excessive control (Guskey, 1998). They also design tasks that enable students with learning difficulties to succeed, reflecting their belief in every learner’s capacity for success (Gibson & Dembo, 1984; Woodcock et al., 2019). Conversely, teachers with low self-efficacy or heightened self-doubt may inadvertently undermine students’ self-efficacy and cognitive development (Bandura, 1993).

These beliefs serve as fundamental indicators of teachers’ behaviors, decisions, and management styles (Fives, 2003). Teachers who perceive external factors as constraining their influence may undervalue their instructional impact. In contrast, those who are confident in their ability to engage unmotivated students view the instructional environment as manageable, which ultimately shapes their professional attitudes, pedagogical strategies, and the overall quality of education (Tschannen-Moran & Woolfolk Hoy, 2001; Woodcock et al., 2019).

### 1.2. Dyscalculia

Dyscalculia, defined as a heterogeneous and persistent cognitive disorder (Sousa et al., 2017), is referred to in the literature by various terms, including “number blindness,” “mathematical disability,” “arithmetic learning difficulty,” and “number sense difficulty” (Doyle, 2010). As one of the specific learning difficulties, dyscalculia is characterized by mathematical difficulties arising from functional impairments in brain regions associated with mathematical cognition (Kosc, 1974). This condition, defined as a significant deficit in mathematical skills, is marked by specific difficulties particularly related to number sense. These difficulties are more pronounced than general impairments in language, memory, or spatial processing (N. C. Jordan et al., 2010). Developmental dyscalculia is conceptualized as a neurodevelopmental disorder characterized by significant and persistent difficulties in learning mathematics that cannot be attributed to low intelligence, sensory impairments, or lack of educational opportunity (Dowker, 2024). This definition underscores the need to differentiate dyscalculia from general mathematical difficulties. Mathematical difficulties can stem from inadequate education, socioeconomic disadvantages, attention problems, or other domain-general cognitive deficits. However, such extrinsic or non-specific factors should not be conflated with developmental dyscalculia (Price & Ansari, 2013). Establishing this conceptual boundary is crucial for accurate identification and the design of educational interventions.

Children with mathematics learning difficulties exhibit various deficits in acquiring mathematical skills. Disruptions in cognitive processes such as working memory, verbal ability, spatial visualization, and numerical symbol comprehension adversely affect mathematical learning (Andersson & Lyxell, 2007; Geary, 2006; Krajewski & Schneider, 2009). Consequently, students with dyscalculia experience deficits across different mathematical domains depending on their age and educational level. These deficits manifest as inadequacies in mental computation, recognition of numerical patterns, execution of basic arithmetic operations, comprehension of area and shape concepts, and skills related to dimension and measurement (Castro et al., 2014).

Students with dyscalculia struggle with associating numbers, comparing magnitudes, and using numbers functionally in daily life due to their difficulty in comprehending the concept of number (Kunwar & Sharma, 2020; Onyishi & Sefotho, 2021). This challenge also manifests in problem-solving processes; such students tend to rely on limited and familiar strategies and struggle to retrieve basic arithmetic facts. Collectively, these difficulties increase the risk of developing mathematics anxiety, which in turn leads to avoidance of mathematics and further hinders the acquisition of fundamental skills (Geary, 2006).

An additional challenge that children with dyscalculia face is memory-related difficulties. These children struggle to learn multiplication tables due to weaknesses in both short-term and long-term memory. They are also known to experience difficulties in determining directionality (e.g., east–west or right–left) (Williams, 2013). Furthermore, students with dyscalculia frequently struggle with money management and understanding various aspects of time. For instance, they may have difficulty learning to read an analog clock and effectively organizing their daily schedules (Burny et al., 2012; Kunwar & Sharma, 2020). Most of these challenges can be mitigated with supplementary support and intensive intervention (Kunwar & Sharma, 2020).

While dyscalculia does not result from inappropriate pedagogical methods, it is evident that accurate knowledge and sound educational practices are essential for successful intervention with these students. Teachers, particularly primary school teachers and mathematics teachers, play a critical role in identifying dyscalculia-related difficulties, especially in the processes of early identification and implementation of effective intervention strategies (Sousa et al., 2017). However, the literature has consistently demonstrated that many teachers lack sufficient knowledge about dyscalculia (Akça & Akgün, 2024; Baldemir et al., 2022; Chinn & Ashcroft, 2006; Dias et al., 2013; Hacısalihoğlu-Karadeniz, 2013; Mutlu et al., 2022; Shalev & Gross-Tsur, 2001; Tennant & Tennant, 2010).

Given that teachers are responsible for the learning processes of all students in their classrooms, it is imperative that they possess knowledge of teaching methods appropriate to students’ individual needs. However, the existing literature indicates that teachers have limited knowledge of effective mathematics instructional methods for children with learning disabilities (DeSimone & Parmar, 2006). This underscores the necessity of equipping teachers with more comprehensive knowledge of dyscalculia and related learning difficulties through both professional development and pre-service training programs. Without such preparation, these students will inevitably lack the support they require and are likely to have adverse learning experiences in mathematics.

### 1.3. Self-Efficacy in Dyscalculia

Dyscalculia is not widely recognized by teachers or educational authorities. However, raising awareness of dyscalculia can benefit these individuals and contribute to improved outcomes (Butterworth et al., 2011). Teachers’ self-efficacy and classroom practices are important factors influencing student achievement and their willingness to work with students with specific learning difficulties (Yakut, 2021). Despite the increasing number of students identified with dyscalculia in schools, little is known about teachers’ knowledge and skills in effectively teaching these students. Mathematics teachers often struggle with intervening for children with dyscalculia because their academic training typically focuses on developing subject matter knowledge. Consequently, they may possess limited knowledge regarding the characteristics of students with dyscalculia and appropriate intervention methods (DeSimone & Parmar, 2006). There is a consensus in the literature that the difficulties experienced by individuals with dyscalculia can be substantially reduced through early identification and appropriate interventions. However, without necessary interventions, the likelihood of these individuals facing more serious adverse consequences increases (Mutlu & Olkun, 2019).

It is important for teachers to create learning experiences that address the abilities and needs of students with learning difficulties, including behavioral challenges (Woodcock et al., 2019). Therefore, it is essential for teachers to be knowledgeable about dyscalculia so that they can develop strategies and methods to support these students. Additionally, informed teachers can enable children with dyscalculia to communicate about their condition with their parents and the broader community (Çam & Mutlu, 2024; Mutlu et al., 2022). Many students with learning difficulties have histories of mathematical failure that lead to feelings of learned helplessness (Gindrich, 2021). Accordingly, mathematics teachers should be able to recognize the symptoms of dyscalculia and know how to intervene appropriately (Tennant & Tennant, 2010).

Teachers play a fundamental role in the education of children with learning disabilities and frequently report feeling inadequately prepared for this responsibility (Brownell et al., 2006). Research has demonstrated that the vast majority of primary school teachers are unaware of dyscalculia and lack the specialized techniques and practical tools necessary to help students overcome these difficulties and realize their potential (Tennant & Tennant, 2010). Moreover, the challenges teachers face may stem from an insufficient willingness to dedicate adequate time to creating content that addresses all students’ needs (Materechera, 2020). Teachers also struggle due to limited knowledge of how to differentiate instructional methods for diverse learning groups within their classrooms (Paulsen, 2013).

Each child with dyscalculia may exhibit different symptoms (Kosc, 1974). Dowker (2009) emphasized that it is difficult to find two students who display identical characteristics of dyscalculia. This variability means that teachers may encounter different profiles in each student with dyscalculia, and an intervention that proves effective for one student may not be effective for another. Therefore, teachers need the capacity to overcome barriers arising from this diversity. According to Bandura (2012), individuals with low self-efficacy readily believe their efforts are futile when confronted with obstacles, whereas those with high self-efficacy tend to find ways to overcome them.

Building on this theoretical perspective, teachers with high self-efficacy invest greater effort in supporting struggling students and demonstrate greater persistence when facing difficulties in the teaching environment. A significant relationship thus exists between teachers’ perceived self-efficacy and their willingness to teach students with learning difficulties (Specht et al., 2016; Yakut, 2021). Indeed, teachers with high levels of self-efficacy assume greater responsibility for the learning of students with special educational needs (Allinder, 1994).

Assessing teachers’ self-efficacy toward dyscalculia is therefore of considerable importance. Individuals’ self-efficacy levels not only affect the amount of effort they exert and the duration of their persistence under adverse conditions but also determine whether they exhibit coping behaviors when confronted with obstacles (Poulou, 2007). Accordingly, there is a significant need to raise awareness about students with mathematics learning disabilities and to enhance both the knowledge and self-efficacy of teachers in addressing their needs (Sağıroğlu et al., 2025).

A review of the literature reveals that scales have been developed to assess teachers’ general self-efficacy (Çapa et al., 2005; Çolak et al., 2017; Karaoğlu, 2019; Hacıömeroğlu & Şahin-Taşkın, 2010), their self-efficacy toward students with learning difficulties (Akıncı et al., 2018; Atik & Düzdemir, 2023; Yulet-Yılmaz et al., 2024), and their self-efficacy related to specific domains (Akıncı et al., 2018; Çocuk et al., 2015; Girgin & Ilgaz, 2022; Yasan-Ak, 2020). Among these specific domains, self-efficacy scales related to special education are noteworthy. For instance, Akıncı et al. (2018) developed a scale to assess music teachers’ self-efficacy perceptions in teaching students with special needs. Çocuk et al. (2015) developed a self-efficacy belief scale for Turkish language teaching. Girgin and Ilgaz (2022) developed a scale to measure the self-efficacy levels of English teachers in the education of gifted students. It is notable that teachers’ self-efficacy regarding dyscalculia has been examined quantitatively through surveys (Chideridou–Mandari et al., 2016; Kunwar & Sharma, 2020; Sousa et al., 2017) and qualitatively through interview protocols (Graves et al., 2018; May et al., 2022; Yulet-Yılmaz et al., 2024). Nevertheless, the need for new and comprehensive scales on this topic persists in the current literature.

The present study differs from previous research in its sample composition, which includes both primary school and mathematics teachers, and in its factor structure, which addresses diagnostic and support processes for dyscalculia more comprehensively. The target population and scope of the scale contribute to the development of measurement instruments that are applicable, valid, and reliable across different contexts, enabling a more holistic assessment of teachers’ competencies related to dyscalculia. The aim of this study is to develop a scale designed to measure the self-efficacy of primary school and mathematics teachers regarding dyscalculia and, subsequently, to provide evidence of validity and reliability to support its use.

## 2. Method

### 2.1. Design

In this study, the guiding steps for scale development proposed by DeVellis (2017) were followed. Accordingly, an initial item pool was generated in the first phase. The items were then refined through Exploratory Factor Analysis (EFA) to identify those to be retained in the scale. Subsequently, the factor structure derived from EFA was tested using Confirmatory Factor Analysis (CFA) to examine the standardized factor loadings of the scale items and the model fit indices.

### 2.2. Item Development and Content Validation

The initial stage of the scale development process followed the steps suggested by DeVellis (2017). Accordingly, the primary aim was to develop a measurement tool designed to assess teachers’ self-efficacy regarding dyscalculia.

#### 2.2.1. Developing the Item Pool

First, a comprehensive literature review was conducted. Theoretical explanations of the concept of self-efficacy, particularly those grounded in Bandura’s (1982) Social Cognitive Theory, were integrated with the literature on dyscalculia. Based on this synthesis, a total of 42 items were generated to measure teachers’ dyscalculia self-efficacy.

#### 2.2.2. Expert Review and Content Validity

The drafted items were reviewed by a panel of seven subject matter and psychometric experts. The panel included two faculty members specializing in Psychometrics, two in Mathematics Education, two in Special Education, and one in Psychology. The experts were asked to evaluate the items in terms of content validity, relevance, clarity, and the appropriateness of the technical language for the target participants. Feedback was collected via an online survey form that included both a rating section for each item and an open-ended comment box. Based on expert evaluations, four items—for example, “I have the necessary knowledge and skills for educational interventions for students with dyscalculia”—were identified as containing overly technical phrasing from the participants’ perspective, which could potentially lead to misinterpretation.

### 2.3. Scale Administration Procedure

Consequently, these four items were removed at this stage, resulting in a pool of 38 items for the initial administration. Subsequent Exploratory Factor Analysis (EFA) indicated that four additional items either failed to load adequately on any factor or exhibited problematic cross-loadings; therefore, they were excluded from the scale. Examples of these items include “I can express mathematical concepts with different representations for students with dyscalculia,” and “I can create appropriate learning environments for children with dyscalculia.” After these item reduction procedures, a final set of 34 items remained. The final items are presented in Appendix A.

Data were collected from two independent samples to examine the validity and reliability of the scale. The first study group was used for the Exploratory Factor Analysis (EFA), whereas the second study group was used for the Confirmatory Factor Analysis (CFA) of the DSES.

### 2.4. Participants and Data Collection

In the Turkish education system, primary school teachers are responsible for teaching all core subjects, including mathematics, at the elementary level. Therefore, both primary school teachers and mathematics teachers were considered relevant target participants for the present study, as both groups are directly involved in mathematics instruction and may encounter students with developmental dyscalculia. To obtain the research data, two independent study groups were recruited using a convenience sampling method. Data were collected electronically through an online survey form distributed primarily via professional teacher networks. The sample included teachers from various provinces across Türkiye, enabling the efficient collection of a dataset sufficient for factor analysis. Participants who completed the data collection instruments incompletely or incorrectly were excluded from the analyses. Survey responses were considered incompletely or incorrectly completed if they met at least one of the following criteria: missing responses, identical responses across all Likert-type items, logically inconsistent answers to reverse-coded or related items.

A total of 515 teachers participated in this study across two independent samples. Overall, 360 participants (69.9%) were women and 155 (30.1%) were men. In terms of professional experience, 161 teachers (31.3%) had 1–5 years of experience, 117 (22.7%) had 6–10 years, 94 (18.3%) had 11–15 years, and 143 (27.8%) had 16 years or more. Regarding subject area, 282 participants (54.8%) were Primary School Teachers and 233 (45.2%) were Mathematics Teachers. The detailed demographic characteristics of each study group are presented below.

Study Group 1 (EFA)

For the Exploratory Factor Analysis (EFA), data were obtained from 273 participants (209 women, 76.5%; 64 men, 23.5%). The distribution of teaching experience was as follows: 1–5 years (n = 73, 26.7%), 6–10 years (n = 51, 18.7%), 11–15 years (n = 54, 19.8%), and 16 years or more (n = 95, 34.8%). In terms of subject area, 149 participants (54.6%) were Primary School Teachers, and 124 participants (45.4%) were Mathematics Teachers.

Study Group 2 (CFA)

For the Confirmatory Factor Analysis (CFA), data were obtained from 242 participants (151 women, 62.40%; 91 men, 37.60%). The distribution of teaching experience was as follows: 1–5 years (n = 88, 36.4%), 6–10 years (n = 66, 27.3%), 11–15 years (n = 40, 16.5%), and 16 years or more (n = 48, 19.8%). In terms of subject area, 133 participants (55.0%) were Primary School Teachers, and 109 participants (45.0%) were Mathematics Teachers.

### 2.5. Measures and Construct Validity Assessment

To assess the psychometric properties of the newly developed DSES, three instruments were administered to the same group of participants. These instruments, along with their conceptual rationale for use, are described below. The Teacher Sense of Efficacy Scale (TSES) was selected to examine convergent validity, as teacher self-efficacy related to general teaching tasks (TSES) is theoretically expected to be strongly and positively associated with self-efficacy related to more specific teaching tasks (DSES). Conversely, the Maslach Burnout Inventory—Education Survey (MBI-ES) was selected to examine discriminant validity, given that self-efficacy, as a positive belief, is theoretically expected to show a moderate negative relationship with burnout, a negative psychological state.

#### 2.5.1. Dyscalculia Self-Efficacy Scale (DSES)

The 34-item Dyscalculia Self-Efficacy Scale (DSES) was developed to assess teachers’ perceived self-efficacy in identifying, supporting, and implementing instructional interventions for students with developmental dyscalculia. The scale uses a five-point Likert-type response format ranging from “Strongly disagree” to “Strongly agree,” indicating the extent to which participants perceive themselves as competent in dyscalculia-related practices. Higher scores reflect higher levels of dyscalculia self-efficacy. The instruction “Please read each item on the scale and mark the option that best represents you” was included in the scale instructions.

#### 2.5.2. Teacher Sense of Efficacy Scale (TSES)

The Teacher Sense of Efficacy Scale (TSES), developed by Tschannen-Moran and Woolfolk Hoy (2001), was administered to examine the convergent validity of the DSES. The scale is grounded in Albert Bandura’s Social Cognitive Theory and consists of 24 items organized into three subdimensions: efficacy for instructional strategies, efficacy for classroom management, and efficacy for student engagement, all of which are designed to assess teacher self-efficacy. [Example item: “To what extent can you use a variety of assessment strategies?”].

Items are rated on a 9-point scale ranging from 1 (Nothing) to 9 (A Great Deal), with higher scores indicating higher levels of teacher self-efficacy. The validity and reliability of the Turkish adaptation of the scale were established by Çapa et al. (2005). In the present study, the Cronbach alpha internal consistency coefficient was 0.965 for the overall scale. The alpha coefficients for the subdimensions—Instructional Strategies, Classroom Management, and Student Engagement—ranged from 0.897 to 0.933.

#### 2.5.3. Maslach Burnout Inventory—Education Survey (MBI-ES)

The Turkish form of the Maslach Burnout Inventory—Education Survey (MBI-ES), originally developed by Maslach et al. (1996), was administered to examine the discriminant validity of the DSES. The inventory consists of 22 items grouped into three subdimensions: Emotional Exhaustion, Depersonalization, and Personal Accomplishment. [Example item: “I feel emotionally drained from my work.”].

All items are rated on a 7-point scale ranging from 0 (never experienced such a feeling) to 6 (experience such feelings every day), with higher scores indicating higher levels of teacher burnout. The validity and reliability of the Turkish adaptation were established by İnce and Şahin (2015). In the present study, the Cronbach alpha internal consistency coefficient for the overall inventory was 0.858. The alpha coefficients for the subdimensions ranged from 0.848 (Personal Accomplishment) to 0.937 (Emotional Exhaustion).

### 2.6. Exploratory Factor Analysis (EFA)

The Principal Axis Factoring (PAF) method was preferred to determine the factor structure and the number of factors to be retained. Decisions regarding factor retention were primarily based on Kaiser’s criterion (Kaiser, 1960). In addition, the suitability of the extracted factor structure for the observed data was further examined using Horn’s parallel analysis (Horn, 1965).

Given the theoretical assumption that the subdimensions of teacher self-efficacy are intercorrelated, the Promax oblique rotation method was employed to allow correlations among factors and to obtain a more meaningful and interpretable factor structure. The final factor structure was evaluated by examining factor loadings, with items loading 0.50 or higher on a single factor and showing no substantial cross-loadings retained for the scale.

### 2.7. Confirmatory Factor Analysis (CFA) and Validation

The structural validity of the developed scale was examined using Exploratory Factor Analysis (EFA) and Confirmatory Factor Analysis (CFA). Convergent validity was evaluated by examining the correlations between DSES scores and scores obtained from the Teacher Sense of Efficacy Scale (TSES), whereas discriminant validity was assessed through correlations between DSES scores and scores from the Maslach Burnout Inventory—Education Survey (MBI-ES). The reliability of the scale was evaluated using Cronbach’s alpha and McDonald’s omega coefficients, with 0.70 adopted as the minimum acceptable threshold.

### 2.8. Results

#### Exploratory Factor Analysis

Data obtained from 273 participants were analyzed using Exploratory Factor Analysis (EFA) conducted with the open-source JASP program (JASP Team, 2024). The suitability of the data for factor analysis was examined using the Kaiser–Meyer–Olkin (KMO) measure and Bartlett’s test of sphericity, and the results indicated that the dataset was appropriate for EFA (KMO = 0.968; χ^2^ = 11,748.101; df = 561; *p* < .001).

Following these preliminary analyses, EFA was performed on the scale. Items with factor loadings below 0.40, as well as items displaying similar loadings across multiple factors (i.e., problematic cross-loadings), were removed from the scale. As a result, the final scale consisted of 34 items and four dimensions, explaining approximately 80% of the total variance. The factor loadings for the retained items are presented in Table 1.

As shown in Table 1, the item–total correlation coefficients of the scale items range from 0.465 to 0.973, while the factor loading values range from 0.581 to 1.009. The internal consistency of the scale was examined using both Cronbach’s alpha (α) and McDonald’s omega (ω) coefficients, which yielded identical values across the dimensions. Accordingly, the composite reliability (CR) values ranged from 0.94 to 0.98, indicating a high level of internal consistency.

The results of the EFA demonstrate that the Dyscalculia Self-Efficacy Scale developed for teachers consists of 34 items and four dimensions. These dimensions are labeled as F1 (Definition of Dyscalculia), F2 (Symptoms of Dyscalculia), F3 (Educational Support for Dyscalculic Children), and F4 (Psychological Support for Dyscalculic Children). In addition, the factor structure of the scale was further examined using parallel analysis based on both real and simulated data, and the results are presented in Table 2.

An examination of the parallel analysis results presented in Table 2 indicates that both the real and simulated data support a four-factor structure for the scale. Specifically, the eigenvalues obtained for each factor in the real data exceeded the corresponding eigenvalues derived from the simulated data. For instance, even the fourth factor, which yielded the lowest eigenvalue among the factors, had a higher eigenvalue in the real data than the average eigenvalue obtained from the simulated data (real data = 0.742; simulated data = 0.605). Taken together, these findings provide strong evidence that the scale demonstrates a four-dimensional structure.

### 2.9. Confirmatory Factor Analysis

The validity of the four-dimensional structure and the scale dimensions obtained for the DSES was further examined using Confirmatory Factor Analysis (CFA). Similar to the EFA stage, the CFA sample consisted of teachers from Mathematics (n = 109; 45.04%) and Classroom Teaching (n = 133; 54.96%). Accordingly, data were collected from a total of 242 participants, including 151 women (62.40%) and 91 men (37.60%).

Prior to CFA, normality analyses were conducted on the collected data. Histogram plots were examined to assess whether the scale items exhibited normal distributions, and Kolmogorov–Smirnov and Shapiro–Wilk tests were performed for each item. The results indicated that the items did not meet the assumption of normality. Therefore, the Unweighted Least Squares (ULS) estimation method was preferred for parameter estimation. The results of the CFA conducted with these data are presented in Table 3.

As shown in Table 3, the standardized factor loadings of the scale items obtained after the CFA ranged from 0.667 to 0.934. In addition, the goodness-of-fit indices of the tested model were calculated as χ^2^ = 1406.541, df = 521, χ^2^/df = 2.689, CFI = 0.992, TLI (NNFI) = 0.992, RMSEA = 0.084 (90% CI = 0.078–0.089), and SRMR = 0.066.

Furthermore, the Average Variance Extracted (AVE) values were computed for each factor to provide additional evidence regarding the scale’s validity. The AVE values were 0.907 for Factor 1, 0.827 for Factor 2, 0.690 for Factor 3, and 0.625 for Factor 4. According to the literature, AVE values exceeding 0.50 are considered acceptable indicators of validity (Cheung et al., 2024; Fornell & Larcker, 1981, p. 46). Taken together, these findings indicate that the scale demonstrates adequate construct validity and can be considered a valid instrument for assessing teachers’ self-efficacy regarding dyscalculia.

### 2.10. Convergent and Discriminant Validity

In addition to the validity evidence reported above for the Dyscalculia Self-Efficacy Scale, further evidence of construct validity was examined through correlation analyses. Specifically, convergent and discriminant validity evidence was obtained by examining the relationships among scores from the Dyscalculia Self-Efficacy Scale, the Teacher Sense of Efficacy Scale, and the Maslach Burnout Inventory—Education Survey. The resulting correlation coefficients are presented in Table 4.

As presented in Table 4, the Pearson correlation coefficients (r) illustrate the relationships among the Dyscalculia Self-Efficacy Scale (DSES), the Teacher Sense of Efficacy Scale (TSES), and the Maslach Burnout Inventory—Education Survey (MBI-ES). The correlations among the subscales of the DSES ranged from r = 0.665 (*p* < .001) to r = 0.810 (*p* < .001), indicating strong positive relationships. In addition, the correlations between the subdimensions and total scores of the DSES and those of the TSES ranged from r = 0.244 (*p* < .001) to r = 0.494 (*p* < .001), suggesting that higher levels of teachers’ self-efficacy regarding dyscalculia are associated with higher levels of general teaching self-efficacy.

By contrast, negative relationships were observed between the subscales and total scores of the DSES and the MBI-ES. These correlation coefficients ranged from r = −.119 (*p* > .005) to r = −0.263 (*p* < .001). However, the total and subscale scores of the DSES were not significantly related to some subdimensions of the MBI-ES. According to Maslach, emotional exhaustion constitutes the core component of burnout. Consistent with this theoretical perspective, a statistically significant, albeit weak, negative relationship was observed between emotional exhaustion and the total DSES score (r = −0.146, *p* < .01). Moreover, personal accomplishment is theoretically the burnout dimension most closely related to self-efficacy. Accordingly, this dimension exhibited statistically significant relationships with both the subdimensions and the total score of the DSES. These findings indicate that as teachers’ dyscalculia self-efficacy decreases, their perceptions of personal accomplishment also decline. Taken together, the results provide strong evidence that the DSES demonstrates adequate convergent and discriminant validity.

## 3. Discussion

The aim of this study is to develop a valid and reliable scale to measure the self-efficacy of primary school teachers and mathematics teachers regarding dyscalculia. As the role of teachers in intervening with students with dyscalculia is critical and self-efficacy affects the quality of the teaching environment (Specht et al., 2016; Yakut, 2021), developing a scale to determine teachers’ self-efficacy with respect to dyscalculia contributes to the literature.

Grounded in Albert Bandura’s Social Cognitive Theory, the Dyscalculia Self-Efficacy Scale (DSES) was developed through a rigorous, multi-stage validation process. Following an extensive review of the dyscalculia literature, an initial pool of items was refined through expert evaluations to ensure content validity and conceptual clarity. Subsequent exploratory and confirmatory factor analyses supported a robust four-factor structure encompassing knowledge of Dyscalculia Symptoms, Providing Psychological Support to Children with Dyscalculia, Diagnosing Dyscalculia, and Providing Support in the Teaching Process. The satisfactory model fit indices and evidence of discriminant validity indicates that the DSES is a psychometrically sound instrument. Taken together, these findings suggest that the scale provides a comprehensive and theoretically grounded measure of teachers’ self-efficacy in addressing dyscalculia, thereby offering a valuable tool for both research and professional development initiatives aimed at improving educational support for students with mathematical learning difficulties.

The findings of this study also extend the existing body of research on teacher self-efficacy and learning difficulties by providing a domain-specific measurement tool that focuses exclusively on dyscalculia. Previous instruments in the literature, such as the general Teacher Self-Efficacy Scale (Tschannen-Moran & Woolfolk Hoy, 2001), have primarily assessed teachers’ overall self-efficacy in general education or their general ability to support students with special educational needs (Akıncı et al., 2018; Girgin & Ilgaz, 2022). In contrast, the DSES uniquely focuses on mathematics-related learning difficulties through the dimensions Dyscalculia Symptoms, Providing Psychological Support to Children with Dyscalculia, Diagnosing Dyscalculia, and Providing Support in the Teaching Process, thereby extending the conceptual framework of teacher self-efficacy in special education. By operationalizing efficacy beliefs specific to dyscalculia, this study not only fills a methodological gap but also provides a foundation for future interventions aimed at enhancing teachers’ readiness and confidence to support students with mathematics learning difficulties.

The internal consistency coefficients of the DSES were found to be very high (Cronbach’s α and McDonald’s ω ≈ 0.98), indicating excellent reliability. Although coefficients exceeding 0.95 may sometimes raise concerns regarding potential item redundancy, such values do not necessarily indicate redundancy when the scale development process includes rigorous content validation and theoretical grounding (DeVellis, 2017; Tavakol & Dennick, 2011). In the present study, extensive precautions were taken to ensure content distinctiveness and prevent item overlap. Specifically, all items were developed based on a comprehensive review of the literature and were evaluated by a panel of subject matter and psychometric experts to establish content validity and conceptual clarity. Expert review is considered a critical step in ensuring that items adequately represent the construct while minimizing redundancy and conceptual overlap (Haynes et al., 1995; Polit & Beck, 2006). Furthermore, the clear factor structure obtained through exploratory and confirmatory factor analyses, along with excellent model fit indices, provides additional evidence that the high internal consistency reflects the homogeneity and coherence of the construct rather than artificial inflation due to redundant items (Brown, 2015; Kline, 2023). Additionally, it is well established that internal consistency coefficients are influenced by the number of items and the strength of their relationships with the underlying construct, and high reliability estimates may naturally occur in well-defined and unidimensional constructs (DeVellis, 2017). Therefore, the high internal consistency coefficients observed in this study are interpreted as evidence of strong construct representation and measurement precision rather than item redundancy.

In the exploratory factor analysis, one item exhibited a factor loading slightly above 1 (λ = 1.009). Although factor loadings are generally expected to range between −1 and 1, values marginally exceeding this range may occasionally occur in exploratory factor analysis due to high communality estimates, strong relationships between items and the underlying construct, or sample-specific estimation characteristics (Hair et al., 2019; Kline, 2023). Importantly, the overall factor structure demonstrated clear interpretability, high explained variance, and strong conceptual coherence. Additionally, all items were carefully developed and evaluated by subject matter experts to ensure content validity and conceptual distinctiveness. Therefore, this result was interpreted as reflecting the strong representation of the construct rather than a methodological artifact or model misspecification.

The total variance explained by the factor structure was approximately 80%, which is relatively high for social science research. This result may be attributed to the strong conceptual coherence among the sub-dimensions, as reflected in the moderate to high inter-factor correlations (r = 0.665–0.810). Although these correlations indicate shared variance, they are theoretically expected because the factors represent closely related facets of a broader higher-order construct—teacher self-efficacy for dyscalculia. For example, teachers’ efficacy in providing instructional support is inherently linked to their ability to recognize dyscalculia symptoms and understand diagnostic processes. Furthermore, all items were carefully developed and evaluated by experts to ensure content validity and conceptual clarity, which likely strengthened the alignment between items and the underlying construct. Consistent with prior research, high explained variance may reflect strong construct representation and structural coherence rather than methodological concerns (Hair et al., 2019).

However, while the factors are clearly integrated, concerns regarding unidimensionality are addressed through robust statistical validation. Both the Exploratory Factor Analysis (specifically Parallel Analysis) and the subsequent Confirmatory Factor Analysis provided clear statistical evidence supporting the four-factor model as the best fit for the data. The strong model fit indices (e.g., CFI = 0.992, TLI = 0.992, SRMR = 0.066) confirmed the construct validity of this four-factor structure. The CFA results obtained in this study are highly consistent with widely accepted guidelines in the methodological literature. According to commonly cited criteria, CFI and TLI values greater than 0.95 indicate excellent model fit, while SRMR values below 0.08 reflect acceptable to good fit (Brown, 2015; Kline, 2023; Hu & Bentler, 1999; Tabachnick & Fidell, 2007). In this context, the observed fit indices (CFI = 0.992, TLI = 0.992, SRMR = 0.066) provide strong evidence that the proposed four-factor model demonstrates a very good fit to the data. However, it should be noted that fit indices may be influenced by model characteristics such as the number of observed variables and overall model complexity; therefore, they should not be interpreted solely based on rigid cut-off criteria (Kenny & McCoach, 2003). Taking together, these findings support the adequacy of the specified measurement model and provide robust empirical evidence for the construct validity of the four-factor structure.

The implications of maintaining this structure, despite the correlations, are significant. This multidimensional approach offers greater practical utility than a single unidimensional score. It allows for a more granular assessment, enabling the identification of specific areas in which teachers may require targeted professional development. For example, a teacher might feel confident in identifying symptoms but lack self-efficacy in providing psychological support (F4). A unidimensional interpretation would obscure this vital nuance, which is essential for designing effective interventions and support systems for educators.

To situate our findings, it is essential to connect them to the broader literature on teacher self-efficacy regarding specific learning difficulties (SLDs). Much of the existing research in this area (e.g., Woodcock et al., 2019; Yakut, 2021) has understandably focused on self-efficacy for SLDs in general, or more commonly on dyslexia. While this research is valuable, it often overlooks the unique cognitive and pedagogical challenges associated with dyscalculia.

This new DSES therefore complements and extends this prior work in a crucial way. It complements generalist instruments (such as the TSES, which we confirmed in our convergent validity analysis) by providing a specialized tool that captures the specific competencies teachers need for dyscalculia—ranging from symptom recognition to differentiated instructional and psychological support.

Furthermore, it extends the field by addressing a significant gap: while the importance of teacher efficacy for dyscalculia has been discussed theoretically, it has historically lacked a dedicated, validated psychometric instrument. By offering this granular, four-factor focus, the DSES provides a more precise diagnostic tool for future research and for the design of targeted professional development, moving beyond broad “SLD self-efficacy” toward the specific needs of mathematics education. It is of great importance for teachers to be able to provide holistic learning experiences by considering not only the academic but also the behavioral and emotional needs of students with learning difficulties (Woodcock et al., 2019). Many students with learning difficulties in mathematics may develop learned helplessness as a result of repeated experiences of failure, which negatively impacts their perceptions of self-efficacy in mathematics (Mutlu, 2024). In this context, it is a critical requirement for mathematics teachers to possess sufficient knowledge and skills to recognize the symptoms of dyscalculia and to develop appropriate intervention strategies for these students (Tennant & Tennant, 2010).

The scale developed in this study was administered to both primary school teachers and mathematics teachers; in terms of scope, it addresses the concept of self-efficacy regarding dyscalculia from a more holistic perspective. The Dyscalculia Symptoms dimension measures teachers’ ability to recognize the cognitive and behavioral symptoms that may be observed in students with dyscalculia. The Providing Psychological Support to Children with Dyscalculia dimension assesses teachers’ capacity to help students cope with feelings such as loss of self-esteem, anxiety, shame, and a sense of failure. The Diagnosing Dyscalculia dimension aims to measure teachers’ levels of knowledge related to identifying and recognizing students at risk of dyscalculia, whereas the Providing Psychological Support to Children with Dyscalculia dimension assesses teachers’ self-efficacy beliefs regarding their ability to make instructional adaptations for students with dyscalculia, determine appropriate strategies, and support the learning process.

The convergent and divergent validity findings obtained in this study provide important insights into understanding the structure of teachers’ professional competencies related to dyscalculia, one of the learning difficulties. The high levels of association among the DSES sub-dimensions indicate that teachers perceive their competencies in recognizing dyscalculia symptoms, participating in the diagnostic process, providing instructional support, and offering psychological support as a holistic structure. To support students experiencing difficulties in mathematics, teachers need to recognize dyscalculia, distinguish its symptoms, and master appropriate instructional strategies (Sousa et al., 2017; Williams, 2013). According to Shulman (1987), the types of knowledge a teacher should possess include subject matter knowledge related to the conceptual and structural characteristics of the discipline; general pedagogical knowledge aimed at facilitating the teaching–learning process; curriculum knowledge concerning the effective use of instructional programs and resources; and knowledge of learners, which involves understanding students’ developmental, cognitive, and affective characteristics. In this respect, the dimensions of the scale correspond to Shulman’s (1987) model of teacher knowledge. Assessing the level of knowledge teachers have regarding this difficulty is of great importance.

The more knowledgeable teachers are about dyscalculia and the more trained they are in appropriate intervention techniques, the more accurately they can support their students (Butterworth et al., 2011). In addition, the positive correlations observed between the DSES and the general teacher self-efficacy scale (TSES) indicate that self-efficacy perceptions related to dyscalculia reflect general teaching self-efficacy and that these two constructs reinforce one another. Teachers’ self-efficacy beliefs directly influence the learning experiences and academic outcomes of students with learning difficulties. When teachers believe they are capable of supporting these students’ learning processes, they tend to employ more patient and effective instructional strategies (A. Jordan et al., 2009). For inclusive education to be implemented effectively, both teachers’ general teaching self-efficacy and their self-efficacy related to specific learning difficulties need to be supported (Özokcu, 2017).

Indeed, research indicates a significant and positive relationship between these two types of self-efficacy; teachers with high general teaching efficacy demonstrate more effective practices in inclusive settings (Kazanopoulos et al., 2022). By contrast, the weak and negative relationships between the DSES and the subscales of the Maslach Burnout Inventory suggest that higher self-efficacy perceptions regarding dyscalculia may reduce teachers’ emotional exhaustion and help maintain their sense of professional efficacy. These findings indicate that strengthening teachers’ knowledge and skills related to dyscalculia may not only enhance instructional effectiveness but also contribute to professional well-being and resilience. Teachers’ self-efficacy levels were found to be significantly and positively associated with their professional well-being, supported by teaching satisfaction, resilience, and emotional balance. It has been emphasized that teachers with high self-efficacy cope more effectively with professional challenges, are more resilient to burnout, and report higher levels of overall professional satisfaction (Li, 2023; Wang et al., 2024). This finding suggests that enhancing knowledge and skills related to dyscalculia may also support teachers’ professional well-being and resilience by increasing their self-efficacy.

Developed in this way, the scale enables the assessment of teachers’ multidimensional self-efficacy perceptions regarding dyscalculia and makes a significant contribution to the existing measurement tools in the field. In addition, to support the validity and reliability of the scale, the Teacher Self-Efficacy Scale (Tschannen-Moran & Woolfolk Hoy, 2001) and the Maslach Burnout Inventory—Educator Form (Maslach et al., 1996) were administered to the same sample group. The high level of agreement obtained indicates that the new scale is a valid and reliable instrument for measuring teachers’ self-efficacy regarding dyscalculia. Although the present study provides evidence for the construct validity and reliability of the DSES, future research should further establish its predictive validity by examining how teachers’ self-efficacy scores are associated with student-level outcomes, such as academic achievement, mathematics anxiety, and referrals for special education support.

The scale developed in this study is based on self-report measurement, in which teachers directly report their own competencies and perceptions through survey responses. Although self-report measures are useful for capturing teachers’ perceptions and beliefs, they may not fully reflect actual classroom practices or instructional competencies. Therefore, the absence of external or behavior-based validation sources (e.g., classroom observations, peer or student evaluations, or student performance indicators) should be acknowledged as a limitation. Future studies could strengthen the evidence for validity by incorporating such data to examine the convergence between perceived and actual efficacy in supporting students with dyscalculia. Moreover, because the DSES assesses perceived self-efficacy rather than objective teaching competence, further research is needed to explore how these beliefs relate to teachers’ actual instructional behaviors and outcomes in inclusive mathematics education.

Another limitation of the present study concerns the characteristics of the research sample. The participants consisted solely of teachers from Türkiye, which restricts the generalizability of the findings to educational settings with different cultural, linguistic, or systemic characteristics. Specifically, because the data were collected using convenience sampling from teachers accessed primarily through professional networks, the sample may not adequately represent the full diversity of the Turkish teacher population, let alone teachers from other educational systems. Therefore, caution should be exercised when generalizing the findings to contexts with differing curricula, teacher training structures, and cultural understandings of dyscalculia. Considering that self-efficacy beliefs are shaped by sociocultural contexts and teaching traditions, cross-cultural validation studies are needed to examine the applicability of the DSES across diverse educational systems. In addition, the gender distribution of the current sample and its regional concentration may not fully represent the broader population of teachers. Future research should aim to include more balanced and diverse samples in terms of gender, region, and teaching context in order to enhance the external validity and international relevance of the scale.

A further limitation concerns the absence of measurement invariance testing across primary school teachers and mathematics teachers. Although the scale was designed to assess dyscalculia-related self-efficacy in both groups, factorial equivalence across teaching specializations was not formally examined. Therefore, potential group comparisons should be interpreted cautiously. Future studies are encouraged to test the stability and equivalence of the factor structure across different teaching disciplines to further strengthen the robustness and generalizability of the DSES.

## 4. Conclusions

In conclusion, this study has successfully developed and validated the Dyscalculia Self-Efficacy Scale (DSES), establishing it as a robust, multidimensional instrument that addresses a significant methodological gap in the field. The scale provides a nuanced tool for assessing the specific self-efficacy beliefs of both in-service and preservice teachers related to supporting students with mathematics learning difficulties.

Building upon this contribution, a critical direction for future research involves the application of the DSES to preservice teacher populations, particularly candidates enrolled in elementary and mathematics education programs. This recommendation is grounded in the theoretical premise that foundational self-efficacy beliefs are significantly shaped during initial teacher education. Assessing the emerging self-efficacy of this cohort with respect to dyscalculia can yield valuable diagnostic insights into their professional preparedness and help identify specific developmental needs prior to their entry into the profession. Consequently, data derived from such studies would be instrumental in informing curricular revisions in teacher education programs, identifying areas requiring greater pedagogical emphasis, and ultimately fostering the early development of strong competence and confidence in inclusive mathematics instruction.

## Figures and Tables

**Table 1:** Factor Loadings for Scale Items.

Items	F1	F2	F3	F4	R_ij_
I16	0.975				0.794
I11	0.959				0.811
I18	0.926				0.805
I10	0.921				0.788
I15	0.901				0.807
I12	0.883				0.775
I13	0.883				0.796
I9	0.861				0.746
I14	0.851				0.759
I17	0.809				0.749
I8	0.581				0.783
I28		0.974			0.743
I29		0.963			0.769
I27		0.954			0.732
I31		0.926			0.744
I26		0.889			0.758
I30		0.889			0.781
I33		0.880			0.792
I34		0.847			0.741
I32		0.748			0.793
I5			1.009		0.682
I6			0.929		0.726
I2			0.921		0.700
I3			0.838		0.771
I4			0.818		0.731
I7			0.686		0.750
I1			0.646		0.736
I24				0.926	0.746
I22				0.873	0.773
I21				0.866	0.705
I23				0.726	0.826
I25				0.722	0.739
I20				0.663	0.787
I19				0.652	0.632
Total Variance	79.8%	60%	10.5%	5.9%	3.4%
Overall Scale Cronbach’s	α = 0.980	α = 0.974	α = 0.979	α = 0.946	α = 0.938
Mc Donald’s	ω = 0.980	ω = 0.974	ω = 0.979	ω = 0.946	ω = 0.937

R_ij_ = Corrected item–total correlation coefficients.

**Table 2:** Parallel Analysis Results.

	Real Data Factor Eigenvalues	Simulated Data Mean Eigenvalues
Component 1	20.748	0.977
Component 2	2.117	0.766
Component 3	1.906	0.677
Component 4	0.742	0.605
Component 5	0.172	0.540

**Table 3:** Factor Loadings Resulting from the CFA Process.

Factor		Items	Estimate	Standardized β	R^2^
Factor 1		I16	0.473	0.860	0.739
AVE	0.722	I11	0.490	0.882	0.778
ω	0.972	I18	0.377	0.759	0.576
α	0.965	I10	0.464	0.851	0.724
Factor Variances	0.180	I15	0.480	0.877	0.770
Estimate	2.137	I12	0.473	0.868	0.753
Standardized β	0.906	I13	0.465	0.857	0.735
		I9	0.473	0.846	0.715
		I14	0.465	0.861	0.741
		I17	0.472	0.871	0.759
		I8	0.429	0.790	0.624
Factor 2		I28	0.562	0.929	0.863
AVE	0.827	I29	0.555	0.915	0.838
ω	0.976	I27	0.565	0.934	0.872
α	0.977	I31	0.553	0.912	0.831
Factor Variances	0.259	I26	0.558	0.912	0.831
Estimate	1.690	I30	0.552	0.907	0.822
Standardized β	0.861	I33	0.515	0.868	0.753
		I34	0.528	0.879	0.772
		I32	0.570	0.927	0.859
Factor 3		I5	0.494	0.806	0.650
AVE	0.690	I6	0.488	0.796	0.634
ω	0.937	I2	0.470	0.798	0.636
α	0.939	I3	0.542	0.873	0.763
Factor Variances	0.265	I4	0.602	0.921	0.848
Estimate	1.667	I7	0.529	0.823	0.677
Standardized β	0.857	I1	0.467	0.770	0.593
Factor 4		I24	0.266	0.790	0.624
AVE	0.625	I22	0.273	0.815	0.664
ω	0.908	I21	0.215	0.667	0.445
α	0.922	I23	0.293	0.862	0.744
Factor Variances	0.081	I25	0.247	0.723	0.523
Estimate	3.374	I20	0.282	0.818	0.670
Standardized β	0.959	I19	0.303	0.826	0.682

**Table 4:** Pearson Correlations.

Variables	1		2		3		4		5		6		7		8		9		10		11		12		13	
1. F1	—																									
2. F2	0.765	***	—																							
3. F3	0.767	***	0.665	***	—																					
4. F4	0.783	***	0.810	***	0.773	***	—																			
5. DSES	0.934	***	0.901	***	0.867	***	0.915	***	—																	
6. SE	0.345	***	0.494	***	0.369	***	0.405	***	0.443	***	—															
7. IS	0.295	***	0.430	***	0.293	***	0.345	***	0.376	***	0.868	***	—													
8. CM	0.244	***	0.333	***	0.226	***	0.259	***	0.295	***	0.808	***	0.828	***	—											
9. TSES	0.306	***	0.437	***	0.307	***	0.346	***	0.385	***	0.944	***	0.949	***	0.936	***	—									
10. EE	−0.120		−0.119		−0.167	**	−0.136	*	−0.146	*	−0.256	***	−0.226	***	−0.287	***	−0.273	***	—							
11. D	−0.121		−0.126		−0.101		−0.113		−0.129	*	−0.294	***	−0.262	***	−0.276	***	−0.298	***	0.639	***	—					
12. PA	−0.158	*	−0.263	***	−0.162	*	−0.198	**	−0.215	***	−0.404	***	−0.404	***	−0.421	***	−0.432	***	0.322	***	0.275	***	—			
13. MBI-SS	−0.161	*	−0.194	**	−0.185	**	−0.181	**	−0.197	**	−0.376	***	−0.347	***	−0.395	***	−0.397	***	0.917	***	0.794	***	0.596	***	—	
ω	0.972		0.976		0.937		0.908		0.981		0.897		0.905		0.933		0.965		0.940		0.888		0.848		0.934	
α	0.965		0.977		0.939		0.922		0.978		0.898		0.906		0.935		0.965		0.937		0.895		0.850		0.925	

* *p* < .05, ** *p* < .01, *** *p* < .001. DSES = Dyscalculia Self-Efficacy Scale Overall Score, SE = Student Engagement, IS = Instructional Strategies, CM = Classroom Management, EE = Emotional Exhaustion, D = Depersonalization, PA = Personal Accomplishment, MBI-SS = Maslach Burnout Inventory—Educators Survey Overall Score.

## Data Availability

The dataset analyzed during the current study and the relevant materials are available from the corresponding author on reasonable request.

## Appendix A. Dyscalculia Self-Efficacy Scale

		**Strongly Disagree**	**Disagree**	**Neutral**	**Agree**	**Strongly Agree**
1.	I know the proposed hypotheses regarding the causes of dyscalculia.					
2.	I know the similarities and differences between dyscalculia and other learning difficulties.					
3.	I understand the differences between a child who is low-achieving in mathematics and a child with dyscalculia.					
4.	I understand the difference between the developmental and acquired types of dyscalculia.					
5.	I have a good knowledge of the concepts and terminology related to dyscalculia.					
6.	I can identify students with dyscalculia in my classroom.					
7.	I know that students with dyscalculia have weak arithmetic skills.					
8.	I know that students with dyscalculia confuse mathematical symbols.					
9.	I know that students with dyscalculia have a poor number sense.					
10.	I am aware that students with dyscalculia can exhibit diverse characteristics.					
11.	I know that students with dyscalculia have difficulty reading an analog clock.					
12.	I know that students with dyscalculia tend to rely on finger counting.					
13.	I am aware that students with dyscalculia have attention deficit.					
14.	I know that students with dyscalculia have difficulty in remembering concepts and forget them easily.					
15.	I know that students with dyscalculia have deficits in mental arithmetic.					
16.	I know that students with dyscalculia have a poor sense of direction and spatial orientation.					
17.	I know that students with dyscalculia feel anxious in maths courses					
18.	I can teach any mathematics topic to students with dyscalculia without difficulty.					
19.	I can design activities that develop number sense in students with dyscalculia.					
20.	I know instructional materials that are more effective for teaching mathematics to students with dyscalculia.					
21.	I can develop instructional materials for a more effective mathematics teaching for students with dyscalculia.					
22.	I can use multiple representations to express mathematical concepts to students with dyscalculia.					
23.	I can design appropriate activities for students with dyscalculia.					
24.	I include activities specifically prepared for students with dyscalculia in my lessons.					
25.	I can enable students with dyscalculia to develop their own strategies for problem-solving.					
26.	I can foster the development of positive attitudes towards mathematics in students with dyscalculia.					
27.	I can reduce the anxiety regarding mathematics of students with dyscalculia.					
28.	I can increase the motivation of students with dyscalculia.					
29.	I can encourage students with dyscalculia to explain how they solved a problem.					
30.	I can encourage students with dyscalculia to use mathematical language.					
31.	I can help students with dyscalculia appreciate the value of mathematics.					
32.	I know how to increase the attention of students with dyscalculia during the lesson.					
33.	I can encourage students with dyscalculia to solve mathematics problems.					
34.	I can help students with dyscalculia enjoy mathematics.					
